# A Simple and Accurate Two-Step Long DNA Sequences Synthesis Strategy to Improve Heterologous Gene Expression in *Pichia*

**DOI:** 10.1371/journal.pone.0036607

**Published:** 2012-05-04

**Authors:** Jiang-Ke Yang, Fang-Yuan Chen, Xiang-Xiang Yan, Li-Hong Miao, Jiang-Hong Dai

**Affiliations:** 1 School of Biology and Pharmaceutical Engineering, Wuhan Polytechnic University, Wuhan, China; 2 School of Life Science, Huazhong University of Science and Technology, Wuhan, China; University of Minho, Portugal

## Abstract

*In vitro* gene chemical synthesis is a powerful tool to improve the expression of gene in heterologous system. In this study, a two-step gene synthesis strategy that combines an assembly PCR and an overlap extension PCR (AOE) was developed. In this strategy, the chemically synthesized oligonucleotides were assembled into several 200–500 bp fragments with 20–25 bp overlap at each end by assembly PCR, and then an overlap extension PCR was conducted to assemble all these fragments into a full length DNA sequence. Using this method, we *de novo* designed and optimized the codon of *Rhizopus oryzae* lipase gene *ROL* (810 bp) and *Aspergillus niger* phytase gene *phyA* (1404 bp). Compared with the original *ROL* gene and *phyA* gene, the codon-optimized genes expressed at a significantly higher level in yeasts after methanol induction. We believe this AOE method to be of special interest as it is simple, accurate and has no limitation with respect to the size of the gene to be synthesized. Combined with *de novo* design, this method allows the rapid synthesis of a gene optimized for expression in the system of choice and production of sufficient biological material for molecular characterization and biotechnological application.

## Introduction

*In vitro* chemical synthesis of long DNA sequences is the foundation of synthetic biology. It was widely used in diverse fields, including codon optimization and *in vitro* functional evaluation of gene, nucleic acid immunity and gene chip preparation, etc. In many cases, a synthesis method is highly desirable to optimize the codon of a gene to achieve high expression levels in heterologous host [1]–[3]. The method for synthesis and assembly of DNA sequences based on oligonucleotides was first described by Agarwal and co-workers [4]. According to their description, the gene synthesis was a typical enzymatic ligation which included 1) chemical synthesis of oligonucleotides, 2) 5'-end phosphorylating the oligonucleotides by T4 polynucleotide kinase, and then 3) ligating the oligonucleotides into the full length gene by T4 ligase. Assembly long DNA sequences from oligonucleotide was first described by Stemmer et al [5]. In this method, a series of oligonucleotides with overlapping sequences covering the complete sequence of both strands of a gene were synthesized, and then progressively generated a full-length molecule by a single assembly PCR (A-PCR). Later, PCR technique was used in gene synthesis, and a series of new methods such as the dual asymmetric PCR and assemble PCR were developed [5], [6]. To facilitate the design and assemble of the oligonucleotides, softwares such as DNAWorks [7], Gene2Oligo [8], and GeMS [9] were developed to make all the oligonucleotides thermodynamically unity. However, such one-step gene synthesis method has its limitations in synthesizing long DNA sequences.

Generally, oligonucleotides with shorter overlapped regions often cause nonspecific mismatches and result in errors such as internal deletions or point mutations of nucleotide. With the increase in length and complexity of DNA sequences, this non-specific match among oligonucleotides becomes more serious and the DNA sequences will be prematurely terminated in PCR reaction. So, in a single batch synthesis reaction, the length of synthesis DNA molecule can only reach less than 600 bp generally [10], [11], [12]. Several strategies such as PCR-based thermodynamically balanced inside-out (TBTO) method for primer designing [13], the sequential ligation and polymerase cycling reaction method [14], PCR-based two-step DNA synthesis (PTDS) method [11], dual asymmetrical PCR and overlap extension PCR (OE-PCR) combined gene synthesis [10], and PCR-based accurate synthesis (PAS) [12] have been developed to overcome these problems and synthesis long DNA sequence. While to make artificial synthesis of long DNA sequence much more widely used in the field of biotechnology, simple and practical gene synthesis methods are continuously sought.

In this study, we developed a simple and accurate two-step gene synthesis technique, in which several DNA fragments (200–500 bp) were firstly synthesized by A-PCR, and then assembled into a full-length gene by OE-PCR. Using this combined A-PCR and OE-PCR method, named AOE, we successfully synthesized a series of genes with different lengths. Here, we described this method and its use in *de novo* designing and optimization of the codons of *Rhizopus oryzae* HU3005 lipase gene *ROL* (810 bp) and *Aspergillus niger* CICC 4009 phytase gene *phy*A (1404 bp) to improve their expression levels in the yeast *Pichia pastoris*.

## Methods

### Strategy for long DNA sequences synthesis

**Figure 1:**
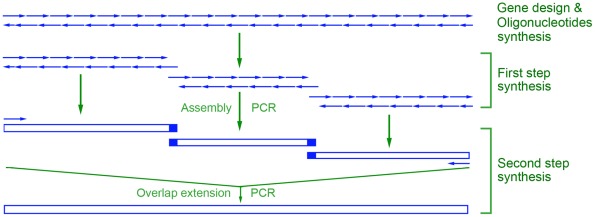
Schema of the two-step gene synthesis method. In the first step, the fragments overlapped with each other was separately assembled by assembly PCR with the outside oligonucleotides as primers; and then, in the second step, were assembled into the full-length gene by overlap extension PCR with 5'- and 3'-end outside primers.

A two-step strategy combining assembly PCR and overlap extension PCR process was developed to synthesize full-length genes (Fig 1). A long DNA sequence was divided into several fragments with size from 200 bp to 500 bp, and overlapped (20–25 nucleotides) at the end of each fragments. To make the thermodynamic properties of each oligonucleotide consistent, and avoid the mismatching among them, we divided a long input DNA sequence into a set of adjacent oligonucleotides representing both DNA strands with the assistant of the Gene2Oligo software [8]. Oligonucleotides were dynamically optimized to ensure both the specificity and the uniform melting temperatures necessary for *in vitro* gene synthesis, and then chemically synthesized by Sangon, Shanghai with the PAGE-grade purity. The nucleotide sequence of oligonucleotides to synthesis *R. oryzae* HU3005 lipase gene *ROL* (GenBank: GQ502721) and *A. niger* CICC 4009 phytase gene *phyA* (GenBank: JN252710) were listed in the (Table S1 and S2).

In the first step, oligonucleotides were assembled into fragments. Assembly PCR reactions were carried out in a 50 μl volume containing 200 µM of each dNTP, 0.1 mM of each oligonucleotide, 1.5 mM MgCl_2_, and 1 U of *Pfu* Turbo DNA polymerase (Stratagene, La Jolla, CA). The PCR thermal cycling was set as a denaturation step at 94°C for 2 min, and 30 cycles of 94°C for 30 s, 55°C for 30 s and 72°C for 1 min, followed by a single incubation at 72°C for 6 min. The products of assembly PCR were re-amplified by another round of PCR using two outer oligonucleotides (Table S1 and S2) in a 50 μl reaction containing 3 μl of assembly PCR mixture, 200 µM of each dNTP, 1 μM of each primer, 1 U of *Pfu* Turbo DNA polymerase in a buffer containing 1.25 mM of MgCl_2_.

In the second step, two or more fragments were assembled into a full-length DNA sequence by overlap extension PCR. A 50 μl PCR mixture contained 200 mM dNTP, 0.1 mM outside primers, and 1 U *Pfu* Turbo. The PCR condition was set as a denaturation step at 94°C for 2 min, and 28 cycles of 94°C 30 s, 55°C 30 s, and 72°C 1 min, followed by an extension step at 72°C for 6 min. The PCR products were then subjected to dA tailing and cloned into pMD18-T simple vector (Takara, Dalian). Three positive clones were selected and sequenced to check their correctness of sequences.

### RNA extraction, original *ROL* and *phyA* genes cloning

To clone the original *ROL* and *phyA* genes, total RNAs from *R*. *oryzae* and *A. niger* were extracted by Trizol reagent (Gibco BRL, Gaithersburg, MD) according to the manufacturer's protocol. The first strand cDNA was synthesis by using the RevertAid First Strand cDNA Synthesis Kit (Fermentas, Hanover, MD). PCR was carried out in a 50 μl reaction containing 200 mM dNTP, 0.1 mM primers, 1.5 mM MgCl_2_, and 1 U of *pfu* DNA polymerase (Takara). The PCR conditions followed were denaturation at 94°C for 5 min, 28 cycles of 94°C for 50 s, 55°C for 50 s and 72°C for 1 min, and final elongation at 72°C for 6 min. The PCR product was cloned into the pMD18-T simple vector (Takara), and then sequenced by Sangon Ltd., Shanghai. The sequence of *R. oryzae* lipase gene (*ROL*) and *A. niger* phtase A gene (*phyA*) were deposited into GenBank with the accession number GQ502721 and JN252710. *R. oryzae* lipase gene *m*-*ROL* was amplified with the primer pairs MROL2 (5′-CT*GAATTC*TCTGATGGTGGTAAGGTTG-3′, *Eco*R I site) and MROLA2 (5′-CT*GCGGCCGC*TTACAAACAGCTTCCTTCGT-3′, *Not* I site). *A. niger phyA* gene was amplified with the primer PhyS (5′-CATGGGTGTCTCTGCCGTTC-3′) and PhyA1 (5′-CGTCAGTATCATGCACTAAG-3′).

### Plasmid construction, transformation and recombinants selection

The full-length genes were digested from pMD18-T simple vector with *Eco*R I and *Not* I enzymes, and then inserted into pPIC9K vector to make the gene fusion expression with α-factor. Enzyme *Sac* I was used to linearize the plasmid for the single crossover with *P. pastoris* genome to generate the methanol-utilized phenotype (Mut^+^). About 5 μg of linearized DNA was mixed with 80 μl of competent cells, and the electroporation was conducted on Gene Pulser (Bio-rad, Richmond, CA) according to the manufacturer's suggestion for *Saccharomyces cerevisiae*. Positive clones were initially selected by MD medium (1.34% yeast nitrogen base, 4×10^−5^% biotin, 2% dextrose) plates and then checked by colony PCR. The insertion copy numbers of the transformants were evaluated by their resistance to Geneticin (G418) as recommended by the company that a single copy of pPIC9K integrated into the *Pichia* genome confers resistance to Geneticin to a level of ∼0.25 mg/ml.

### Fermentation and protein inducible expression

The process for protein inducible expression was conducted mainly according to the description of Yang et al [15]. Briefly, a single colony of recombinant was picked and inoculated into 50 ml BMGY medium (1% yeast extract, 2% peptone, 100 mM potassium phosphate buffer with pH 6.0, 1.34% yeast nitrogen base, 4×10^−5^% biotin, 1% glycerol), and grew at 28°C in a shaking incubator (250 rpm) until the culture reached an OD_600_ of 3.0. The cells were harvested and transferred into 50 ml BMMY medium (1% yeast extract, 2% peptone, 100 mM potassium phosphate buffer pH 6.0, 1.34% yeast nitrogen base, 4×10^−5^% biotin, and 0.5% methanol) to obtain a cell suspension with OD_600_ = 1.0. The expression of enzyme was induced by methanol at a final concentration of 0.5% added every 24 h, and the activity was checked at all the time intervals.

### Protein content and activity determination and assays

Protein content of the fermentation broth was determined by the Bradford method [16]. To check the protein profile in fermentation broth by SDS-PAGE, equal volumes of supernatant of the fermentation broth of different recombinants (methanol induced expression for 48 h) were collected and precipitated by 40% NH_4_SO_4_ and re-solved in equal volume of TE buffer (pH 7.5). After dialysis in TE buffer overnight, the protein profile was checked by SDS-PAGE. Lipase activity was quantified at pH 7.5 by free fatty acid titration with 50 mM NaOH after incubation in a thermostated vessel for 10 min. The assay mixture consisted of 5 ml 50 mM Tris–HCl buffer, 50 mM NaCl, 4 ml emulsified olive oil and 1 m1 enzyme solution. One unit (U) of the activity was defined as the amount of enzyme liberating 1 micromole of fatty acid per min at 45°C. The method to check the phytase activity is based on the principle that inorganic phosphate is released from the substrate phytate under defined assay conditions and the activity of phytase was determined mainly according to the description by Gizzi et al [17]. Briefly, phytase activity assay was carried out in 1.0 mL volume at 37°C for 10 min in 200 mM sodium acetate buffer (pH 5.5) containing 2 mM sodium phytate. The released inorganic orthophosphates were quantified spectrophotometrically by the molybdate-blue reaction [18]. One unit of phytase activity was defined as the amount of enzyme required to release 1 μmol phosphate per min under assay condition.

## Results

### Gene design

**Figure 2:**
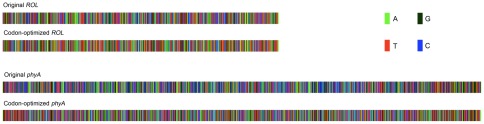
Sequence comparison between the original and the codon optimized genes. Dots in figure represent the same nucleotides between original and optimized genes. (A) Original and optimized *ROL* gene, and (B) Original and optimized *phyA* gene.

**Figure 3:**
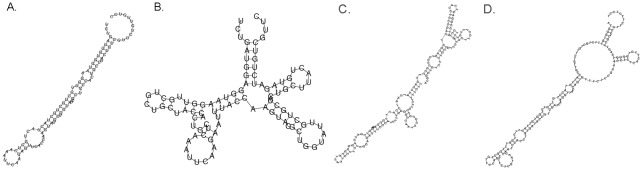
Secondary structure of the first 100-150 bp of original and optimized mRNA generated by the software RNAfolder. A. Structure of original *ROL* mRNA with the MFE is −27.3 kcal/mol and (B) structure of codon optimized ROL mRNA with the MFE is −24.5 kcal/mol; (C) Structure of original *PhyA* mRNA with the MFE is −67.8 kcal/mol and (D) structure of codon optimized *PhyA* mRNA with the MFE is −45.9 kcal/mol.

Because of the significant difference in codon usage bias between *R. oryzae*, A. niger and *P. pastoris*, the usage frequency of most of the codon what *ROL* and *PhyA* genes encoded are less frequently used in *P. pastoris* (Fig S1 and S2). To achieve a high-level expression of foreign genes in Pichia, factors such as codon usage and complexity of secondary structure of mRNA were considered. 1) Based on the native amino acid sequence of ROL and PhyA, the codons of the these genes were optimized by replacing the codons predicted less frequently used in *Pichia* with the frequently used ones; 2) In order to prevent the exhaustion of the tRNA, four most frequently used amino acid (Lys, Asp, Glu, Asn) have not been fully optimized; 3) though the evenly distribution of A, T, G and C could efficiently deduce the complexity of the secondary structure of mRNA, high frequency codons were not always chosen to make G, C, A and T evenly distribution in the gene in order to eliminate AT- or GC-rich motifs and keep GC content of the synthetic gene at 45–60% (Fig 2, Fig S3). The complexity of mRNA secondary structure and the minimal free energy (MFE) were calculated by RNAfold software [19]. After codon optimization, the complexity of the RNA secondary structure and the minimal free energy of designed *R*. *oryzae ROL* gene and *A. niger phyA* gene have significantly changed from the original -235.26 kcal/mol and -531.99 kcal/mol to −229.01 kcal/mol −450.56 kcal/mol, respectively (Fig 3).

### Assembly PCR and overlap extension PCR (AOE) combined two-step gene assembly

**Figure 4:**
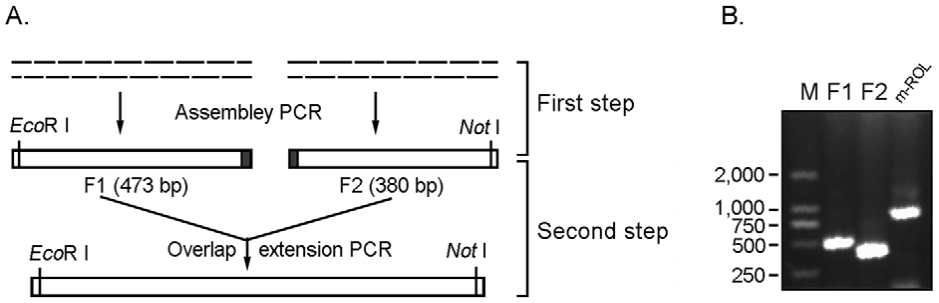
Schema of the two-step *ROL* gene synthesis method. Full length *ROL* gene was firstly divided into two overlapped fragments, F1 (473 bp) and F2 (380), and were assembled by assembly PCR separately; Then F1 and F2 was assembled into full length *ROL* gene by overlap extension PCR (A). (B) PCR products of F1, F2 and full-length *ROL* gene.

**Figure 5:**
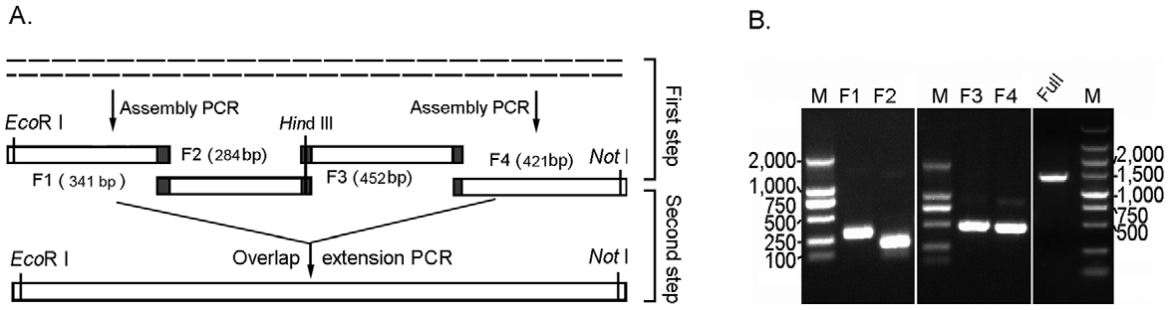
Schema of the two-step *phyA* gene synthesis method. Full length *phyA* gene was divided into four fragments (F1, F2, F3 and F4) with the end overlapped with each other and assembled by assembly PCR separately. In the second step, these four fragments were assembled into a full length gene by overlap extension PCR (A). (B) PCR products of F1, F2, F3, F4 and full-length *phyA* gene.

According to the size of the synthesis gene, *ROL* gene (810 bp) was divided into two fragments, and *phyA* gene (1404 bp) was divided into four fragments. The steps of two-step gene synthesis were shown by the flowchart in figure 4A and figure 5A. In the first step, assembly PCR was conducted to assemble the oligonucleotides covering both strands of DNA molecule into the fragments. This step was similar to the general one-step assembly PCR gene synthesis method described by Stemmer et al. [5]. In the second step, an overlap extension PCR with the end sequence of the full-length gene as the primers was conducted to assemble these fragments into the full length gene (Fig 4B and Fig 5B), and the details were described in the “Materials and Methods” section.

### Expression of the original and codon optimized genes in *P. pastoris*

**Figure 6:**
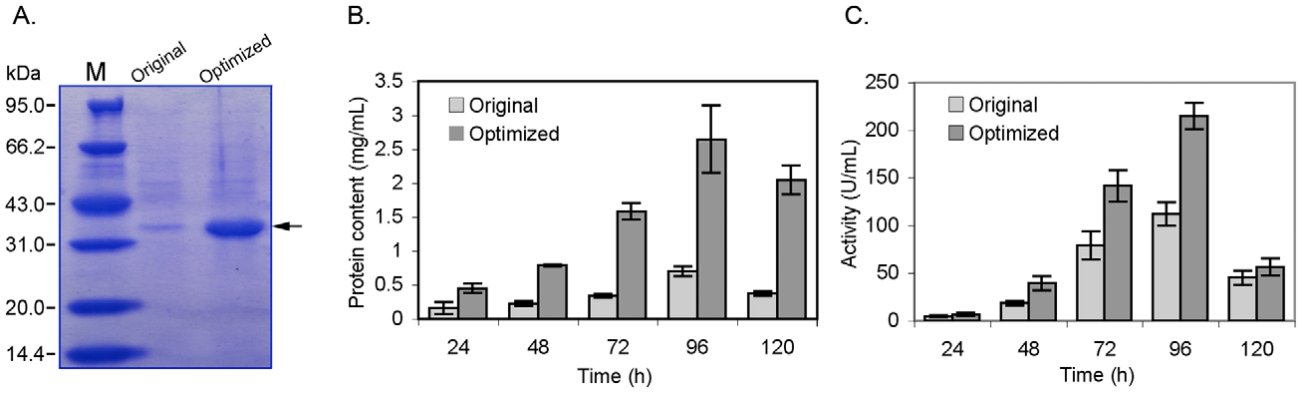
Expression profiles of yeast recombinants carrying original and codon optimized *ROL* gene (*ROL-syn*). (A) SDS-PAGE analysis of protein in fermentation broth after methanol induced for 48 h. About 20 μL protein solution per sample was loaded into the gel stained by coomassie blue G-250 after running. Protein content (B) and lipase activity (C) of fermentation broth of yeast recombinants carrying original and codon optimized *ROL* genes.

**Figure 7:**
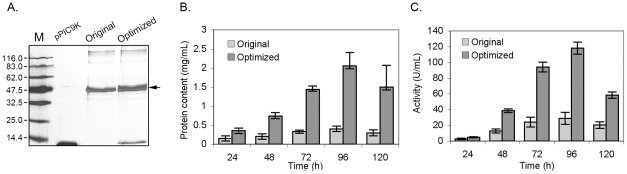
Expression profiles of yeast recombinants carrying original and codon optimized *PhyA* gene (*phyA-syn*). (A) SDS-PAGE analysis of protein in fermentation broth after methanol induced for 48 h. About 20 μL protein solution per sample was loaded into the gel which gone to silver stain after running; Protein content (B) and phytase activity (C) of fermentation broth from yeast recombinants carrying original and codon optimized *phyA* genes.

To evaluate the effect of the codon optimization, plasmids carrying original or new codon optimized gene were transformed and expressed in yeast an fermentation broth were checked by SDS-PAGE gel after induction. And enzyme activities were measured and calculated (Fig 6 and Fig 7). According to the SDS-PAGE gel, both the original and the codon optimized gene were efficiently expressed in yeast, respectively (Fig 6A and Fig 7A). A significant improvement in gene expression level was observed on the codon optimized genes. After inducible expression for 96 h, the enzyme production and activity curves show that both the activity and the protein level in the supernatant of gene-optimized recombinants reached the maximal levels. To *ROL* optimized gene recombinants, the maximal protein content and lipase activity reached 2.7 mg/mL and 220.0 U/ml, while the recombinants carrying original gene had only 0.4 mg/mL and 118.5 U/ml, respectively. To *phyA* gene recombinants, the maximal protein content and phytase activity reached 2.2 mg/mL and 122 U/mL, respectively, while the protein content and activity of the recombinants carrying the original *phyA* had only 0.35 mg/ml and 25.6 U/mL, respectively.

## Discussion

Problems like nonspecific mismatch between oligonucleotides and the truncated sequences caused by premature termination of PCR reaction are commonly confronted by the method that synthesizes a DNA sequence in a batch. With the increase of the length of DNA sequence and structural complexity, these problems become more serious and also enlarge the risk of premature termination of DNA molecules. In order to overcome these problems, in this study, we adopted a two-step strategy combining assemble PCR and overlap extension PCR to synthesize long DNA sequences. In this method, the number of oligonucleotides in one reaction was significantly reduced, thus the chance for pre-mature termination, nucleotides deletion and mutation in synthesized DNA sequences decreased accordingly, and also the successful rate was dramatically increased. Different from other two-step methods previously described [10]–[13], assembly PCR and overlap extension PCR method developed in this study is simple and mature, and can be easily mastered by researchers (Table 1).

**Table 1:** Comparison of different methods used for gene synthesis.

Items	Single-step method [5]	PTDS method [11]	Two-step method [10]	PAS method [12]	AOE method (This study)
Steps for gene synthesis	Sigle step: assembly PCR	Two steps: Successive PCR & Overlap exptension PCR	Two steps: Dual asymmetrical PCR & Overlap extension PCR	Two steps: Dual asymmetrical PCR-like & Overlap extension PCR	Two steps: Assembly PCR & Overlap extension PCR
Softeware used for oligo designing	NM ^1^	NM	NM	DNAWorks & GeMS	Gene2Oligo
Length of oligos (nt)	40	60	50	∼60	30-50
Purity of the oligo	No purification	PAGE	Desalt purification	PAGE	PAGE
Polymerase	Taq	Pyrobest Taq	pfu	Pfu or pyrobest Taq	Pfu
Accuracy or error rate	NM (76% gene functional expressed)	Error rate is 1.26/1000	3/4 clones contain one and three single base deletions	Error rate is ∼1/1000	Eerror rate <1/1000
Editing	Not mentioned	OE-PCR	T7 endonuclease I treatment	OE-PCR	Not recommended

1, NM: Not mentioned;

Current oligonucleotide synthesis technologies always produce by-products that are either prematurely terminated, or contain internal deletions in the sequence. This is the main reason to introduce gaps in synthesized DNA sequences. With the increase of the length of oligonucleotide, the frequency of errors increases, and also the percentage of correct synthesized DNA sequences dramatically decreases as more oligonucleotides are used. Although PAGE purified or even HPLC purified oligonucleotides could reduce these errors to some extent, but the recovery ratio of these oligonucleotides dramatically decreases with the increase of length, and the mutation problem can only be solved by reducing the length of oligonucleotides used to assemble a gene [10]. Compared with the length of oligonucleotides used in other one-step or two-step synthesis method in which the oligonucleotides used generally longer than 60 bp [6], [10]–[12], the length of the oilgonucleotides used in this study are shorter than 50 bp (Table 1, Table S1 and S2), thus significantly reduced the gaps and the chance of point mutant. Using DNA polymerase without proofreading function such as rTaq is another reason to introduce point mutations into the synthesized DNA sequences. As what we can imagine, DNA polymerase with high fidelity could efficiently reduce this type of mutation, and thus enzyme such as *pfu* was recommended and used in this study.

Generally, a laborious and time exhausting post-synthesis nucleotide editing process is needed to eliminate mutations and gaps in synthesized DNA sequence. While in our two-step strategy, this nucleotide editing step is not necessary. Combined with PEGA-grade oligonucleotides and high fidelity DNA polymerase used in our method, the gaps could be efficiently eliminated and the mutation ratio can be reduced down to 0.1%–0.05% (Table 1). To get a 100% accurate clone, we generally sequenced 2–3 colonies more and selected the accurate one.

Parameters included AT-rich regions, GC-rich regions, the overall nucleotide composition and the general codon usage were previously described to affect gene expression or even cause a premature transcription termination in yeast [20], [21]. To enhance the expression level of gene, codon optimization with high frequency codon is often used but not always used the highest frequency codon. Hosts like *Pichia* are A/T codon preferential, and the most frequently used codons are generally A/T biased (http://www.kazusa.or.jp/codon/). So, during the course of gene design, we tried to make G, C, A and T distributed in a gene evenly to avoid complex secondary structure of mRNA or pre-termination due to the A/T rich domain in yeast cells. According to our results, even distribution of G, C, A and T also reduced the complexity and enhanced the MFE, which enhanced expression (Fig 3).

In this study, a simple and efficient two-step gene synthesis method was developed and successfully used in *ROL* and *phyA* gene synthesis. We believe this strategy to be of special interest as it allows the rapid synthesis of a gene optimized for expression in the system of choice and production of sufficient amounts of biological materials for molecular characterization and biotechnological application. The enzyme production of the recombinants carrying optimized *ROL* and *phyA* genes may be further improved under the batch-induced mode with a tighter control of parameters such as pH, methanol concentration and aeration during the fermentation process.

## Supporting Information

Figure S1 Codon usage frequency of the original *ROL* gene (A) and codon optimized *ROL* gene (B).
(TIF)

Figure S2 Codon usage of the original *phyA* gene (A, B) and codon optimized *phyA* gene (C, D).
(TIF)

Figure S3 **Sequence comparison between the original and the codon optimized genes.** Dots in figure represent the same nucleotides between original and optimized genes. (A) Original and optimized *ROL* gene, and (B) Original and optimized *phyA* gene.
(TIF)

Table S1 Oligonucleoitides for *ROL* gene synthesis.
(DOC)

Table S2 Oligonucleoitides for *phyA* gene synthesis.
(DOC)
