# Supplementary figures and images for: A Simple and Accurate Two-Step Long DNA Sequences Synthesis Strategy to Improve Heterologous Gene Expression in Pichia
Source: PLoS One. 2012 May 4;7(5):e36607. doi: 10.1371/journal.pone.0036607 (PMC3344903; doi:10.1371/journal.pone.0036607)

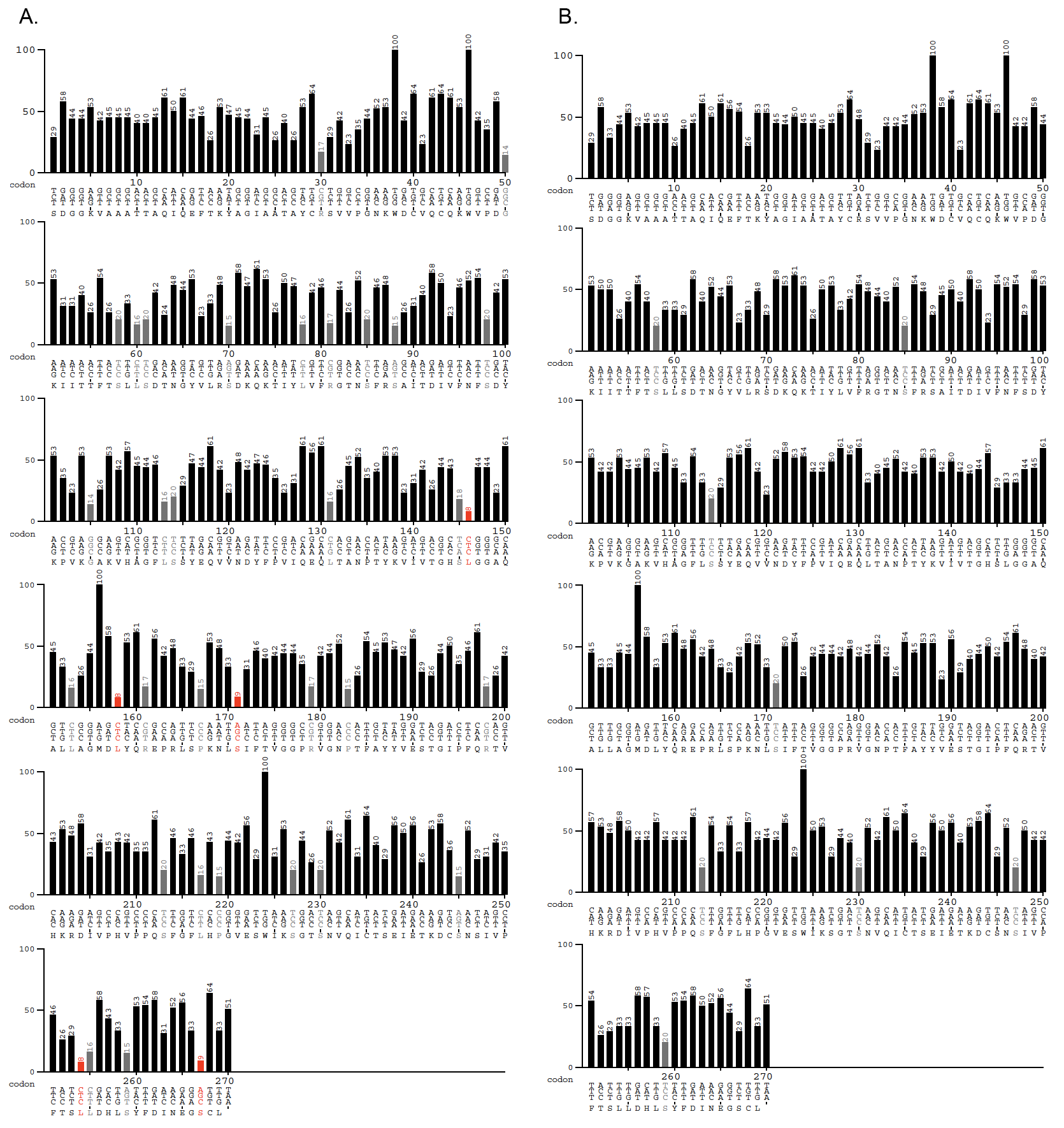

Supplement: Figure S1 — Codon usage frequency of the original ROL gene (A) and codon optimized ROL gene (B). (TIF) [file pone.0036607.s001.tif]

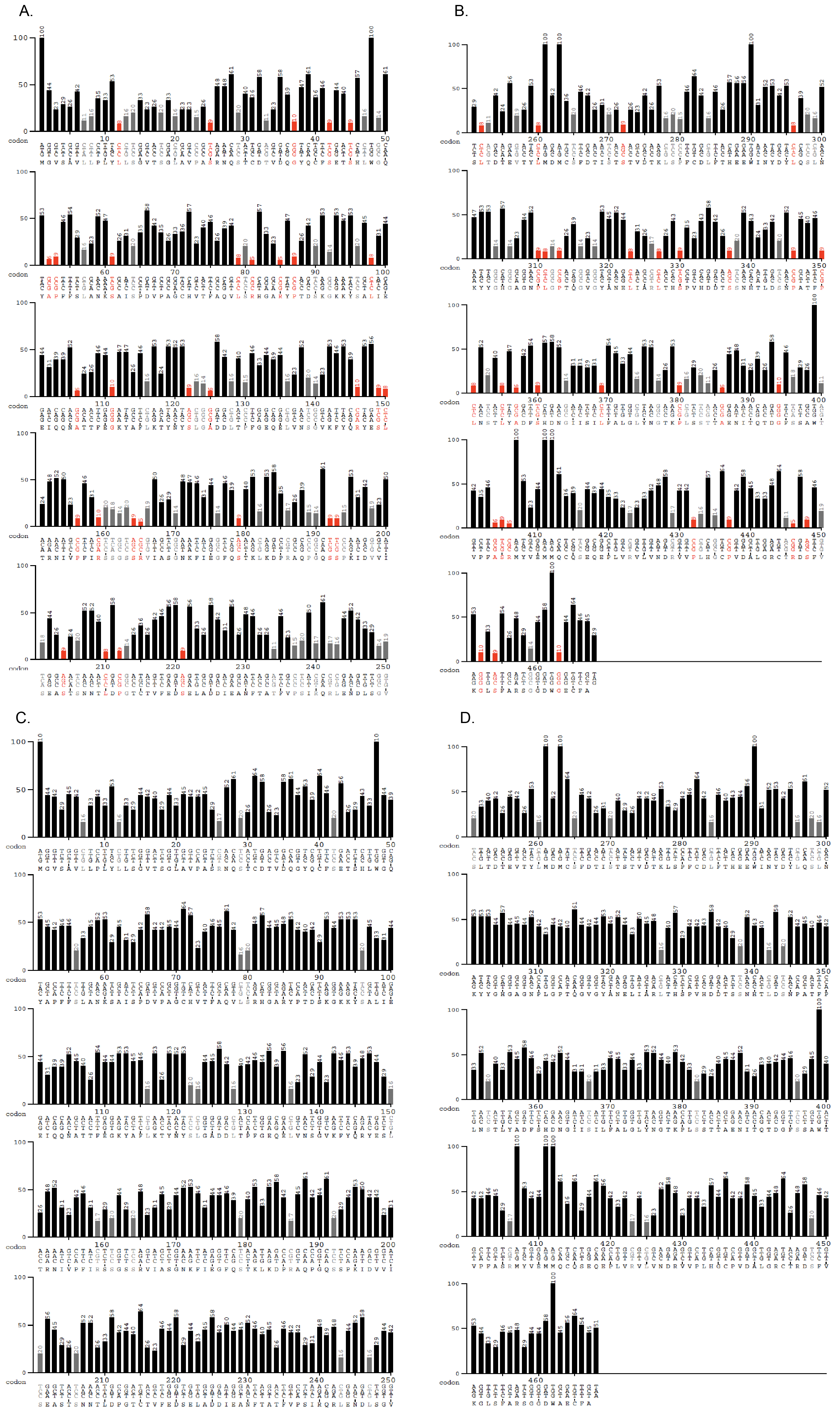

Supplement: Figure S2 — Codon usage of the original phyA gene (A, B) and codon optimized phyA gene (C, D). (TIF) [file pone.0036607.s002.tif]

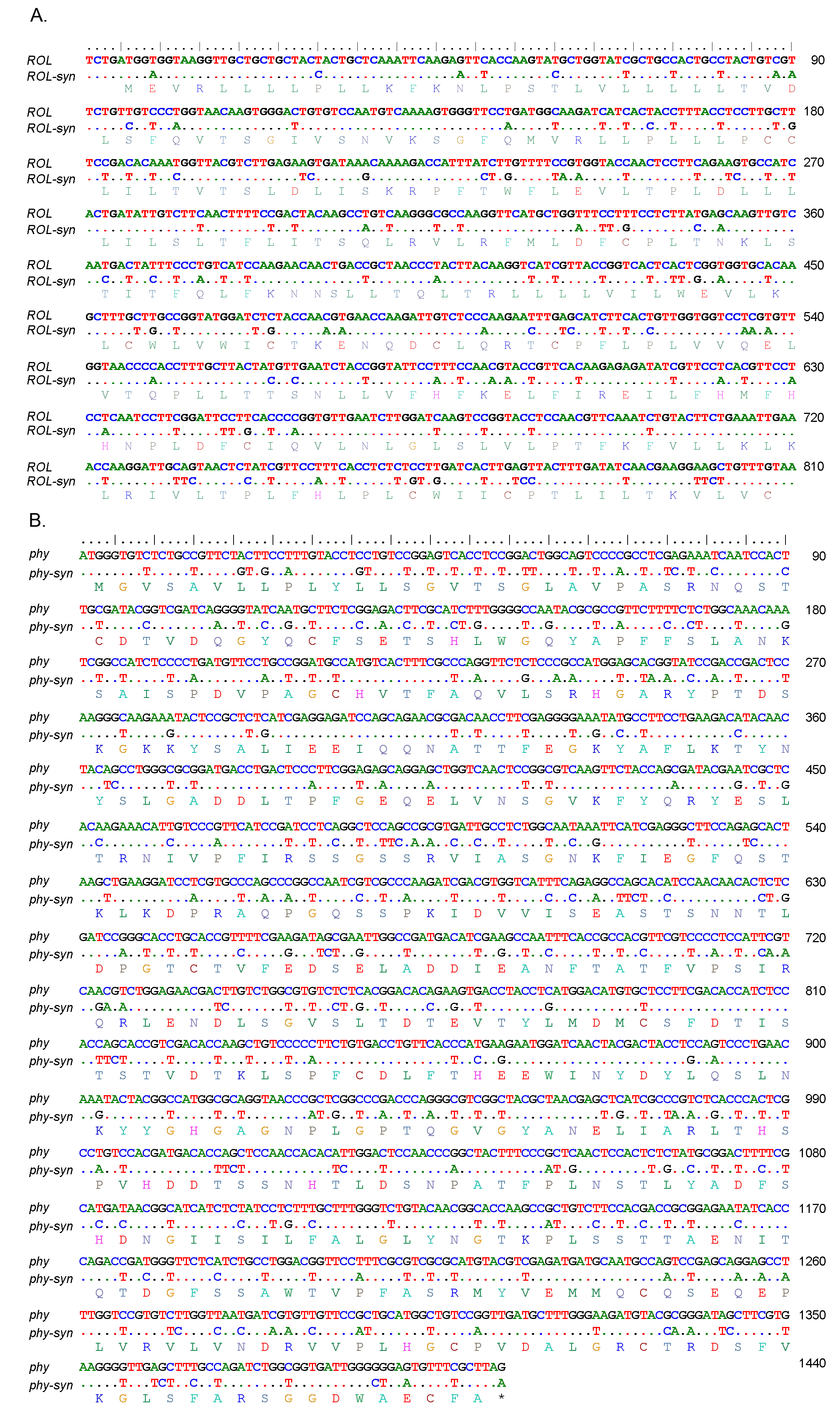

Supplement: Figure S3 — Sequence comparison between the original and the codon optimized genes. Dots in figure represent the same nucleotides between original and optimized genes. (A) Original and optimized ROL gene, and (B) Original and optimized phyA gene. (TIF) [file pone.0036607.s003.tif]
