# Supplementary material for: A Simple and Accurate Two-Step Long DNA Sequences Synthesis Strategy to Improve Heterologous Gene Expression in Pichia
Source: PLoS One. 2012 May 4;7(5):e36607. doi: 10.1371/journal.pone.0036607 (PMC3344903; doi:10.1371/journal.pone.0036607)
Supplement: Table S1 — Oligonucleoitides for ROL gene synthesis. (DOC) [file pone.0036607.s004.doc]

Table S1 Oligonucleoitides for *ROL* gene synthesis

| ID | Sequence (5’-3’) | Number of nucleotide |
| --- | --- | --- |
| 1R0 | GAGAATTCtatgttccaatgta | 22 |
| 1F0 | tacattggaacataGAATTCTCTGATGGAGGTAAGGTT | 38 |
| 1R22 | CAGTGGTAGCAGCAGCAACCTTACCTCCATCA | 32 |
| 1F38 | GCTGCTGCTACCACTGCTCAAATTCAAGAATTTACCA | 37 |
| 1R54 | GCAATACCAGCGTACTTGGTAAATTCTTGAATTTGAG | 37 |
| 1F75 | AGTACGCTGGTATTGCTGCTACTGCTTACTGT | 32 |
| 1R91 | CTGGAACGACAGATCTACAGTAAGCAGTAGCA | 32 |
| 1F107 | AGATCTGTCGTTCCAGGTAACAAGTGGGATTGT | 33 |
| 1R123 | CCACTTTTGACATTGGACACAATCCCACTTGTTAC | 35 |
| 1F140 | GTCCAATGTCAAAAGTGGGTTCCAGATGGTAAGATT | 36 |
| 1R158 | AGGAAGTAAAAGTGGTAATAATCTTACCATCTGGAAC | 37 |
| 1F176 | ATTACCACTTTTACTTCCTTGTTGTCTGATACTAACG | 37 |
| 1R195 | CAGATCTCAAGACGTAACCGTTAGTATCAGACAACA | 36 |
| 1F213 | GTTACGTCTTGAGATCTGATAAGCAAAAGACCATTTAC | 38 |
| 1R231 | TTAGTACCTCTAAAAACCAAGTAAATGGTCTTTTGCTTAT | 40 |
| 1F251 | TTGGTTTTTAGAGGTACTAACTCCTTTAGATCTGCTATT | 39 |
| 1R271 | AAAAGTTAAAGACAATATCAGTAATAGCAGATCTAAAGGAG | 41 |
| 1F290 | ACTGATATTGTCTTTAACTTTTCTGATTACAAGCCAGTT | 39 |
| 1R312 | GAACCTTAGCACCCTTAACTGGCTTGTAATCAG | 33 |
| 1F329 | AAGGGTGCTAAGGTTCATGCTGGATTTTTGTCC | 33 |
| 1R345 | GACAACTTGTTCGTAAGAGGACAAAAATCCAGCAT | 35 |
| 1F362 | TCTTACGAACAAGTTGTCAACGATTACTTTCCAGTT | 36 |
| 1R380 | CAGTCAATTGTTCTTGAATAACTGGAAAGTAATCGTT | 37 |
| 1F398 | ATTCAAGAACAATTGACTGCTAACCCAACTTACAAGG | 37 |
| 1R417 | AGAATGACCAGTAACAATAACCTTGTAAGTTGGGTTAG | 38 |
| 1F435 | TTATTGTTACTGGTCATTCTTTGGGAGGTGCTCAAG | 36 |
| 1R455 | ataataaataagggcaatttAAGCTTGAGCACCTCCCAA | 39 |
| 1R471 | CTTaaattgcccttatttattat | 23 |

(Table S1 continued)

| ID | Sequence (5’-3’) | Number of nucleotide |
| --- | --- | --- |
| 2R0 | GCTTctccaccgatatac | 18 |
| 2F0 | gtatatcggtggagAAGCTTTGTTGGCTGGTATGG | 35 |
| 2R18 | GGTTCTCTTTGGTACAAATCCATACCAGCCAACAAA | 36 |
| 2F35 | ATTTGTACCAAAGAGAACCAAGATTGTCTCCAAAGAAC | 38 |
| 2R54 | AACGGTAAAAATGGACAAGTTCTTTGGAGACAATCTT | 37 |
| 2F73 | TTGTCCATTTTTACCGTTGGTGGTCCAAGAGTTG | 34 |
| 2R91 | GCAAAGGTTGGGTTACCAACTCTTGGACCACC | 32 |
| 2F107 | GTAACCCAACCTTTGCTTACTACGTCGAATCTACT | 35 |
| 2R123 | GTTCTTTGAAATGGAATACCAGTAGATTCGACGTAGTAA | 39 |
| 2F142 | GGTATTCCATTTCAAAGAACTGTTCATAAGAGAGATATTGT | 41 |
| 2R162 | GTGGTGGAACATGTGGAACAATATCTCTCTTATGAACA | 38 |
| 2F183 | TCCACATGTTCCACCACAATCCTTTGGATTTTTGCAT | 37 |
| 2R200 | CAAGATTCAACACCTGGATGCAAAAATCCAAAGGATT | 37 |
| 2F220 | CCAGGTGTTGAATCTTGGATTAAGTCTGGTACTTCCA | 37 |
| 2R237 | CAGAAGTACAAATTTGAACGTTGGAAGTACCAGACTTAATC | 41 |
| 2F257 | ACGTTCAAATTTGTACTTCTGAAATTGAAACTAAGGATTGTTC | 43 |
| 2R278 | TAAATGGAACAATGGAGTTAGAACAATCCTTAGTTTCAATTT | 42 |
| 2F300 | TAACTCCATTGTTCCATTTACCTCTTTGTTGGATCATTT | 39 |
| 2R320 | TTCGTTAATATCAAAGTAGGACAAATGATCCAACAAAGAGG | 41 |
| 2F339 | GTCCTACTTTGATATTAACGAAGGTTCTTGTTTGTAAGCGG | 41 |
| 2R361 | gtaaggctttccGCGGCCGCTTACAAACAAGAACC | 35 |
| 2R380 | CCGCggaaagccttac | 16 |
