# Supplementary material for: A Simple and Accurate Two-Step Long DNA Sequences Synthesis Strategy to Improve Heterologous Gene Expression in Pichia
Source: PLoS One. 2012 May 4;7(5):e36607. doi: 10.1371/journal.pone.0036607 (PMC3344903; doi:10.1371/journal.pone.0036607)
Supplement: Table S2 — Oligonucleoitides for phyA gene synthesis. (DOC) [file pone.0036607.s005.doc]

Table S2 Oligonucleoitides for *phyA* gene synthesis

| ID | Sequence (5’-3’) | Number of nucleotide |
| --- | --- | --- |
| 1R0 | ACACCCATGAATTCacgt | 18 |
| 1F0 | acgtGAATTCATGGGTGTTTCTGCTGTTCTGTTGC | 35 |
| 1R18 | CCAGACAACAGGTACAATGGCAACAGAACAGCAGAA | 36 |
| 1F35 | CATTGTACCTGTTGTCTGGTGTTACTTCTGGTTTGGC | 37 |
| 1R54 | TACGAGAAGCTGGAACAGCCAAACCAGAAGTAACA | 35 |
| 1F72 | TGTTCCAGCTTCTCGTAACCAATCCACCTGTGA | 33 |
| 1R89 | TAACCTTGATCGACGGTATCACAGGTGGATTGGT | 34 |
| 1F105 | TACCGTCGATCAAGGTTACCAGTGTTTCTCCGAAAC | 36 |
| 1R123 | ACCCCACAAGTGAGAGGTTTCGGAGAAACACTGG | 34 |
| 1F141 | CTCTCACTTGTGGGGTCAGTACGCTCCATTCTTC | 34 |
| 1R157 | AGACTTGTTAGCCAAGGAGAAGAATGGAGCGTACTG | 36 |
| 1F175 | TCCTTGGCTAACAAGTCTGCTATCTCTCCAGATGTTCC | 38 |
| 1R193 | TGACATGACAACCAGCTGGAACATCTGGAGAGATAGC | 37 |
| 1F213 | AGCTGGTTGTCATGTCACTTTCGCTCAAGTTCTGT | 35 |
| 1R230 | TCTAGCACCATGTCTGGACAGAACTTGAGCGAAAG | 35 |
| 1F248 | CCAGACATGGTGCTAGATACCCAACTGACTCTAAGG | 36 |
| 1R265 | CAAAGCGGAGTACTTCTTACCCTTAGAGTCAGTTGGGTA | 39 |
| 1F284 | GTAAGAAGTACTCCGCTTTGATCGAGGAGATCCAGCA | 37 |
| 1R304 | CTCAAAGGTAGTAGCGTTCTGCTGGATCTCCTCGAT | 36 |
| 1F321 | GAACGCTACTACCTTTGAGGGTAAGTACGCTTTCCTG | 37 |
| 1R340 | GGGAGTAGTTGTAGGTCTTCAGGAAAGCGTACTTACC | 37 |
| 1F358 | AAGACCTACAACTACTCCCTGGGTGCTGATGACCT | 35 |
| 1R377 | TGTTCACCGAATGGAGTCAGGTCATCAGCACCCA | 34 |
| 1F393 | GACTCCATTCGGTGAACAGGAACTGGTCAACTCTGG | 36 |
| 1R411 | TCTCTGGTAGAACTTGACACCAGAGTTGACCAGTTCC | 37 |
| 1F429 | TGTCAAGTTCTACCAGAGATACGAGTCTCTGACCAGA | 37 |
| 1R448 | GGATGAATGGGACGATGTTTCTGGTCAGAGACTCGTA | 37 |
| 1F466 | AACATCGTCCCATTCATCCGTTCTTCCGGTTCTTCCA | 37 |
| 1R485 | TACCAGAAGCGATGACTCTGGAAGAACCGGAAGAAC | 36 |
| 1F503 | GAGTCATCGCTTCTGGTAACAAGTTCATCGAGGGT | 35 |
| 1R521 | TCAACTTAGTGGACTGGAAACCCTCGATGAACTTGT | 36 |
| 1F538 | TTCCAGTCCACTAAGTTGAAGGATCCACGTGCTCA | 35 |
| 1R557 | GAGAGGATTGACCTGGTTGAGCACGTGGATCCT | 33 |
| 1F573 | ACCAGGTCAATCCTCTCCAAAGATTGACGTTGTCATC | 37 |
| 1R590 | acacagaAAGCTTCGGAGATGACAACGTCAATCTTTG | 37 |
| 1F610 | TCCGAAGCTTtctgtgtaataccgccaaggtcta | 34 |
| 1F627 | tagaccttggcggtatt | 17 |

(Table S2 continued)

| ID | Sequence (5’-3’) | Number of nucleotide |
| --- | --- | --- |
| 2R0 | GAAGCTTcagggattaggata | 21 |
| 2F0 | tatcctaatccctgAAGCTTCTACCTCCAACAACACCT | 38 |
| 2R21 | GTACAAGTACCTGGATCCAAGGTGTTGTTGGAGGTA | 36 |
| 2F38 | TGGATCCAGGTACTTGTACCGTCTTCGAGGATTCTGA | 37 |
| 2R57 | TCAATGTCATCAGCCAACTCAGAATCCTCGAAGACG | 36 |
| 2F75 | GTTGGCTGATGACATTGAGGCTAACTTCACTGCTAC | 36 |
| 2R93 | GTCTGATAGATGGAACGAAGGTAGCAGTGAAGTTAGCC | 38 |
| 2F111 | CTTCGTTCCATCTATCAGACAGAGACTGGAGAACGATC | 38 |
| 2R131 | TCAAGGAAACACCAGACAGATCGTTCTCCAGTCTCT | 36 |
| 2F149 | TGTCTGGTGTTTCCTTGACTGACACCGAGGTTACC | 35 |
| 2R167 | ACACATGTCCATCAGGTAGGTAACCTCGGTGTCAG | 35 |
| 2F184 | TACCTGATGGACATGTGTTCCTTCGACACCATCTC | 35 |
| 2R202 | GTGTCAACGGTAGAAGTGGAGATGGTGTCGAAGGA | 35 |
| 2F219 | CACTTCTACCGTTGACACTAAGTTGTCTCCATTCTGTG | 38 |
| 2R237 | CTCGTGAGTGAACAGGTCACAGAATGGAGACAACTTA | 37 |
| 2F257 | ACCTGTTCACTCACGAGGAATGGATCAACTACGACTAC | 38 |
| 2R274 | CTTGTTCAGGGATTGCAGGTAGTCGTAGTTGATCCATTC | 39 |
| 2F295 | CTGCAATCCCTGAACAAGTACTACGGTCATGGTGC | 35 |
| 2R313 | ACCCAATGGGTTACCAGCACCATGACCGTAGTA | 33 |
| 2F330 | TGGTAACCCATTGGGTCCAACTCAAGGTGTTGG | 33 |
| 2R346 | CAATCAACTCGTTAGCGTAACCAACACCTTGAGTTGG | 37 |
| 2F363 | TTACGCTAACGAGTTGATTGCTAGACTGACTCATTCTCCA | 40 |
| 2R383 | AAGAAGTGTCATCGTGAACTGGAGAATGAGTCAGTCTAG | 39 |
| 2F403 | GTTCACGATGACACTTCTTCCAACCACACTCTGGA | 35 |
| 2R422 | AAGTAGCTGGGTTGGAATCCAGAGTGTGGTTGG | 33 |
| 2F438 | TTCCAACCCAGCTACTTTCCCATTGAACTCCACT | 34 |
| 2R455 | GAGAGAAATCAGCGTACAAAGTGGAGTTCAATGGGA | 36 |
| 2F472 | TTGTACGCTGATTTCTCTCACGACAACGGTATCATCT | 37 |
| 2R491 | CCAAAGCGAACAAGATGGAGATGATACCGTTGTCGT | 36 |
| 2F509 | CCATCTTGTTCGCTTTGGGTTTGTACAACGGTACTAAGC | 39 |
| 2R527 | GCAGTGGTAGAGGACAATGGCTTAGTACCGTTGTACAAAC | 40 |
| 2F548 | CATTGTCCTCTACCACTGCTGAGAACATCACCCAGAC | 37 |
| 2R567 | CAGAGGAGAAACCGTCAGTCTGGGTGATGTTCTCA | 35 |
| 2F585 | TGACGGTTTCTCCTCTGCTTGGACTGTTCCATTCG | 35 |
| 2R602 | CTCAACGTACATACGAGAAGCGAATGGAACAGTCCAAG | 38 |
| 2F620 | CTTCTCGTATGTACGTTGAGATGATGCAATGCCAATCT | 38 |
| 2R640 | AACCAATGGTTCTTGCTCAGATTGGCATTGCATCAT | 36 |
| 2F658 | GAGCAAGAACCATTGGTTCGTGTTCTGGTCAACG | 34 |
| 2R676 | TGCAATGGAACGACTCTATCGTTGACCAGAACACG | 35 |
| 2F692 | ATAGAGTCGTTCCATTGCATGGTTGTCCAGTTGATGC | 37 |
| 2R711 | TCTGGTACATCTACCCAAAGCATCAACTGGACAACCA | 37 |
| 2F729 | TTTGGGTAGATGTACCAGAGATTCCTTCGTTAAGGGTT | 38 |
| 2R748 | CCAGATCTAGCGAAAGACAAACCCTTAACGAAGGAATC | 38 |
| 2F767 | TGTCTTTCGCTAGATCTGGTGGTGATTGGGCTGAA | 35 |
| 2R786 | GCCGCTTAAGCAAAACATTCAGCCCAATCACCA | 33 |
| 2F802 | TGTTTTGCTTAAGCGGCCGCttaagctgatggc | 33 |
| 2F819 | gccatcagcttaaGCG | 16 |
